# A Case of Amyand’s Hernia With Abscess Managed by Two-Stage Surgery: Elective Hernia Mesh Repair Following Emergency Laparoscopic Appendectomy

**DOI:** 10.7759/cureus.68486

**Published:** 2024-09-02

**Authors:** Yoshiyuki Chiba, Daisuke Usuda, Takeshi Yamamoto, Shingo Kawano, Hiroyuki Sugo

**Affiliations:** 1 Department of General Surgery, Juntendo University Nerima Hospital, Tokyo, JPN; 2 Department of Emergency and Critical Care Medicine, Juntendo University Nerima Hospital, Tokyo, JPN

**Keywords:** inguinal hernia mesh repair, complicated acute appendicitis, abscess formation, two-stage surgery, amyand’s hernia

## Abstract

An 82-year-old man presented to our emergency department with a bulge in the right groin and worsening pain that had been present for one week. An abdominal computed tomography scan revealed fluid collection within a right inguinal hernia and a thickened appendix within the hernia sac. The patient underwent an emergency laparoscopic appendectomy under a diagnosis of Amyand’s hernia with peri-appendicular abscess. During surgery, the incarcerated appendix was pulled back into the abdominal cavity from the hernia sac, and the perforated appendix was resected. For drainage of the abscess, a drain tube was laparoscopically placed into the hernia sac through the internal inguinal ring. Considering the risk of mesh infection and wound infection, the patient underwent appendectomy alone but not hernia repair at this time. Two months later, Lichtenstein repair using mesh was performed as a second-stage procedure. For Amyand’s hernia with abscess, this type of two-stage strategy may avoid the surgical site infection, and the use of mesh in a second procedure would minimize the possibility of hernia recurrence, unlike previously reported cases treated by concomitant appendectomy and hernia repair.

## Introduction

The first example of successful appendectomy in a patient with an inflamed appendix contained within an inguinal hernia sac was described by Amyand [1]. Amyand’s hernia is now defined as a very rare inguinal hernia that contains the appendix, regardless of whether it is normal, inflamed, or perforated. It represents 0.4-0.6% of all hernias [2], and cases complicated by acute appendicitis or perforated appendix account for 0.1% [3]. Although both appendectomy and hernia repair are necessary for the treatment of Amyand’s hernia with appendicitis, the optimal treatment strategy is still controversial because the use of mesh for hernia repair carries a risk of mesh infection. On the other hand, preoperative diagnosis is difficult unless imaging modalities are used. For this reason, most reported cases of Amyand’s hernia have been diagnosed intraoperatively, necessitating simultaneous appendectomy and hernia repair as a one-stage operation [4]. Against this background, due to the high incidence of surgical site infection (SSI), hernia repair without using mesh has been the predominant approach.

Here, we present the first reported example of two-stage surgery, including elective hernia mesh repair following emergency laparoscopic appendectomy based on preoperative diagnosis, in order to avoid the risk of mesh infection and reduce the likelihood of hernia recurrence.

"This article was previously presented at the 22nd Annual Congress of Japanese Hernia Society on May 25, 2024."

## Case presentation

**Figure 1:**
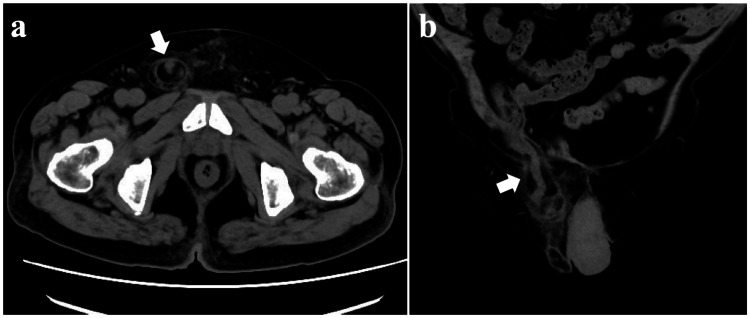
Abdominal CT scan one month ago: (a) axial view and (b) coronal view. Abdominal CT reveals the right inguinal hernia containing the appendix (arrows), without any sign of inflammation. CT: computed tomography.

An 82-year-old man was referred to our hospital with suspected inguinal hernia in view of persistent right groin discomfort and swelling. His medical history included open radical prostatectomy for prostate cancer five years previously. An abdominal computed tomography (CT) scan revealed a right inguinal hernia containing the appendix but no concomitant appendicitis. Accordingly, elective hernia repair was planned two months ahead under a diagnosis of Amyand’s hernia with a non-inflamed appendix (Figure 1).

**Figure 2:**
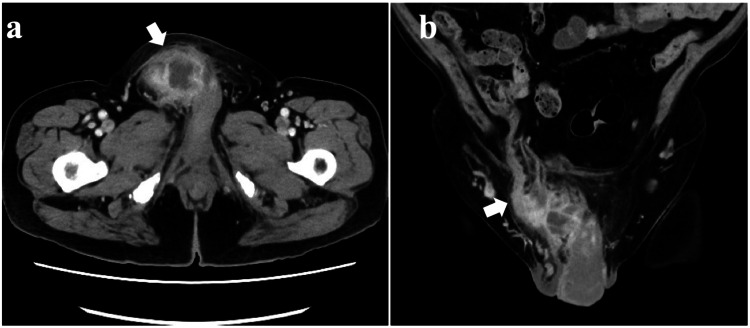
Abdominal contrast-enhanced CT on admission: (a) axial view and (b) coronal view. The abdominal CT demonstrates heterogeneous fluid collection within the right inguinal canal (a; arrow) and a thickened appendix within the hernia sac (b; arrow). CT: computed tomography.

However, one month later, the patient presented to the emergency department with a bulge in the right groin and severe worsening inguinal pain. He was afebrile, but laboratory data included WBC 10,800/μl and CRP 20.8 mg/dl, indicating a severe inflammatory reaction. Physical examination revealed redness in the groin area and swelling the size of a chicken egg. The pain was so severe that the patient had difficulty walking. A CT scan of the abdomen and pelvis revealed fluid collection within the right inguinal hernia and a thickened appendix within the right hernia sac (Figure 2). 

On the basis of these findings, we made a diagnosis of Amyand’s hernia with peri-appendicular abscess formation due to a perforated appendix. The patient therefore underwent emergency laparoscopic appendectomy. 

**Figure 3:**
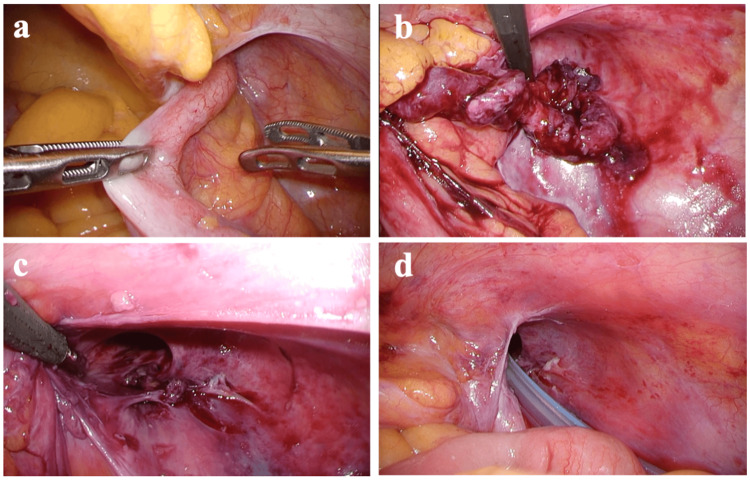
Intraoperative findings. (a) The appendix was incarcerated within the right inguinal hernia. (b) The appendix was removed from the hernia sac. (c) Purulent material was present in the right inguinal hernia orifice. (d) A drain was inserted into the hernia sac.

Intraoperatively, the appendix was seen to be incarcerated within the right inguinal internal ring (Figure 3).

**Figure 4:**
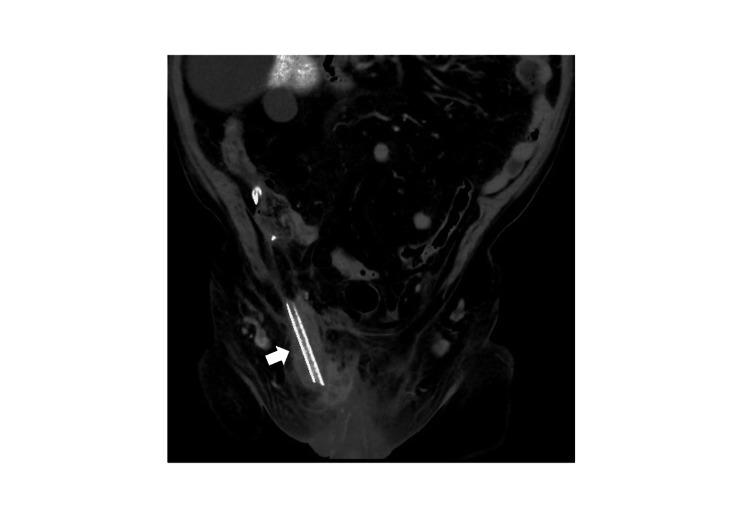
Abdominal CT after surgery. A drain was placed in the hernia sac (arrow). CT: computed tomography.

After laparoscopic adhesion release, the incarcerated appendix was pulled back into the abdominal cavity from the hernia sac, and the perforated appendix was resected. To drain the abscess in the hernia sac, a drainage tube was placed extending from the inguinal internal ring into the hernia sac (Figure 4).

At this time, only appendectomy with abscess drainage was performed, without hernia repair, to avoid the risk of postoperative mesh and wound infection. Hernia repair was scheduled for later as a second-stage procedure, considering the high recurrence rate of non-prosthesis hernia repair. 

The patient was subsequently treated with antibiotics and discharged from the hospital on the 13th postoperative day. Two months after the emergency surgery, we performed Lichtenstein repair using mesh for the right inguinal hernia. Intraoperatively, no unusual adhesion was found in the inguinal canal, and the procedure was performed uneventfully. The patient was discharged from the hospital on the first postoperative day and is currently doing well 10 months after surgery without any hernia recurrence. 

## Discussion

Amyand’s hernia is a rare form of inguinal hernia, and its pathophysiology is not fully understood. It is still controversial whether appendicitis is the initial cause or whether its herniation causes compression at the level of the deep inguinal ring, causing blood flow alterations, bacterial overgrowth, and thus the development of appendicitis [5]. In this case, a preoperative diagnosis of Amyand’s hernia with a non-inflamed appendix was made by CT scan one month before surgery, then appendicitis occurred one month later. Preoperatively, a CT scan can accurately identify the hernia and aid the decision over whether conservative or surgical treatment is warranted [5]. However, it is difficult to diagnose Amyand’s hernia preoperatively, unless radiologic modalities are used [5].

Physical examination will usually reveal swelling in the right groin, pain, and tenderness. Depending on the vermiform appendix’s situation (normal, inflamed, perforated, or gangrenous), other symptoms that may appear are fever, vomiting, gastrointestinal symptoms, and bowel obstruction, but this connection is inconsistent because the neck of the hernia will usually prevent the spread of inflammation and limit peritoneal irrigation, making the clinical picture less acute than expected [6]. Also, typical symptoms of acute appendicitis, such as initial epigastric pain settling later in the right iliac fossa, nausea, vomiting, and anorexia, may also be seen in patients with an Amyand’s hernia [7]. Consequently, Amyand’s hernia generally presents as an incarcerated or strangulated hernia, so most patients undergo emergency hernia repair first, without any suspicion of the presence of appendicitis. As a result, an inflamed or perforated appendix will be revealed within the inguinal canal, and an appendectomy will be performed subsequently. This may be the reason why almost all patients with this condition undergo simultaneous hernia repair and appendectomy as a one-stage procedure.

With regard to treatment, Losanoff and Basson have proposed a classification for Amyand’s hernia, thus setting a therapeutic framework (Table 1) [8]. 

**Table 1:** Losanoff and Basson classification of Amyand’s hernia.

Classification	Description	Management
Type 1	Normal appendix in an inguinal hernia	Hernia reduction or mesh replacement
Type 2	Acute appendicitis in an inguinal hernia with no abdominal sepsis	Appendectomy, primary non-prosthetic hernia repair
Type 3	Acute appendicitis in an inguinal hernia with abdominal and abdominal wall sepsis	Laparotomy, appendectomy, and primary non-prosthetic hernia repair
Type 4	Acute appendicitis in an inguinal hernia with abdominal concomitant pathology	As with type 3, disease-specific management is added

The classification system is based on the recommended surgical treatment, which varies according to the type of Amyand’s hernia. They recommend laparotomy, appendectomy, and primary non-prosthetic hernia repair for patients with acute appendicitis in an inguinal hernia with abdominal and abdominal wall sepsis, classified as type 3. On the other hand, one-stage surgery for Amyand’s hernia with abscess presents two problems: First, there is a high risk of SSI because hernia repair may create a contaminated surgical field due to inguinal abscess formation. Ko et al. have demonstrated that the rate of SSI in Amyand’s hernia with abscess is as high as 24%, compared with 16% for Amyand’s hernia without abscess [9]. Second, there is a risk of hernia recurrence in the event of non-prosthesis hernia repair. In Amyand’s hernia with abscess, there is a need to avoid the use of mesh in order to prevent mesh infection. Including the present case, only 24 cases of Amyand’s hernia with abscess formation have been reported in the English literature with detailed information on the patients, and a review of these 24 cases revealed that 23 (96%) of them, except for the present case, were treated by concomitant appendectomy and hernia repair in a single stage [2,7,10]. In addition, in 22 (97%) of these 23 cases, mesh was avoided due to contamination. Although the recurrence rate of hernia in these patients who underwent non-mesh repair was unclear, a previous study of hernia repair in a total of 260 patients demonstrated that recurrence rates in the mesh repair group were significantly lower than for non-mesh repair [11].

In this case, we were able to diagnose Amyand’s hernia with abscess preoperatively using CT, and thus, prior laparoscopic appendectomy was performed without hernia mesh repair to avoid the risk of SSI. This is the first reported example of two-stage surgery, including elective hernia mesh repair followed by emergency laparoscopic appendectomy. Laparoscopic surgery is less invasive than open surgery and allows detailed observation of the intraperitoneal state, making it more effective for confirming the diagnosis of hernia [11]. In addition, by using laparoscopic surgery, we were able to perform appendectomy and irrigation drainage with a good visual field and percutaneous opening of the inguinal canal. As a result, no adhesion was found during the second surgery, and hernia repair using mesh was performed as the Lichtenstein technique as with the initial surgery. In addition, this two-stage strategy may have helped to shorten the operation time in the first procedure. 

## Conclusions

In conclusion, it is difficult to diagnose Amyand’s hernia preoperatively unless radiologic modalities are used, and treatment strategy is still controversial, especially in Amyand’s hernia with abscess formation. When it is diagnosed preoperatively using CT, this two-stage strategy might reduce the likelihood of SSI, and the use of mesh in the second procedure would reduce the possibility of hernia recurrence. 
